# Amount of external CME in groups of specialties: a nation-wide survey among Finnish doctors

**DOI:** 10.1186/1756-0500-2-265

**Published:** 2009-12-28

**Authors:** Arja Helin-Salmivaara, Mira Kajantie, Jukka Vänskä, Hannu Halila, Taina Autti, Juha P Turunen, Amos Pasternack

**Affiliations:** 1 Unit of General Practice, Hospital District of Helsinki and Uusimaa, Helsinki, Finland; 2 Unit of Education, Finnish Medical Association, Helsinki, Finland; 3 Helsinki Medical Imaging Center, Department of Radiology, University Hospital of Helsinki, Helsinki, Finland; 4 Unit of Education, Finnish Medical Society Duodecim, Helsinki, Finland; 5 Association for Medical Continuous Professional Development in Finland, Helsinki, Finland

## Abstract

**Background:**

Continuing medical education (CME) is an integral part of continuing professional development and a prerequisite for good quality in health care. We aimed to describe and analyse the number of days spent in formal CME outside the workplace by specialty among Finnish doctors of working age.

**Findings:**

The number of days in formal CME outside the workplace in 2005 reported by specialists was obtained from an annual postal survey, conducted by the Finnish Medical Association in March 2006, of all working-age doctors. Those who had attained their specialist degree before 2005 were included in the study. The 49 specialties were re-categorised into 15 groups. The mean reported number of days and 95% confidence intervals were calculated. Differences were analysed by Poisson regression adjusted for relevant covariates.

The response rate to the question about CME was 70.2% (7,374) among specialists. The median age (interquartile range) of the respondents was 49 years (from 44 to 55 years), and 51.7% (3,810) were female. The mean reported number of days in CME was 8.8 (95% CI 8.7-9.0). Neurologists and surgery specialists participated in CME the most frequently (10.3 and 10.4 days) and ophthalmologists the least (7.6 days). In comparison with anaesthesiology and intensive care specialists, most specialists reported having significantly more formal CME, and no group reported having less.

**Conclusions:**

Significant variation was observed, and we therefore suggest studies seeking to account for this variation.

The results have originally been published in Finnish in the Finnish Medical Journal.

## Findings

Continuing medical education (CME) is an integral part of continuing professional development (CPD), which is considered, in turn, as a prerequisite for good quality in health care. In several countries, the amount of formal CME each specialist has to attend yearly is regulated by a recertification system. In some countries CME is voluntary, based on the assumed development needs of individual doctors or their working organisations. In Finland, no mandatory external requirements exist regarding the amount of formal CME or its content. According to legislation passed in 2004, health-care employers have to ensure that employees, including doctors, participate sufficiently in CME in order to maintain and develop their professional skills. No absolute amount is mentioned. However, the Finnish Medical Association recommended in 1999 a yearly minimum of ten days of formal external CME.

Participation has been surveyed annually by the Finnish Medical Association since the early years of the 2000s' and the mean number of days has varied between 7.6 and 8.8 among those who had participated in any CME [1-3]. Learning and development needs and consequently, implications for the quantity or form of various professional development activities, including CME, may vary between specialities.

Little is known about actual realisation of CME among various specialists in either the regulatory or the voluntary system. We aimed to describe and analyse the number of days spent in 2005 in formal CME outside the workplace according to specialty among Finnish doctors of working age.

## Methods

### Setting

Public health services are available to all permanent residents, regardless of their financial situation, and services are mainly financed from tax revenues. The doctor density is high: there is one doctor of working age per 301 inhabitants.

Basic medical education lasts at least six years. In order to receive full authorisation, Finnish doctors have to undergo two years of additional training in primary health care. Of Finnish doctors, 63% are specialists and 15% are currently undergoing specialist training. The previously used 92 specialisation programmes were replaced in 1999 by 49 new specialties. Most of the specialties require six years' training.

### Survey

The study is based on the March 2006 results of an annual postal questionnaire, conducted by the Finnish Medical Association, of non-retired doctors of working age (n = 17,768), irrespective of their membership status [3]. Among other issues, the doctors were asked to report the number of days they had spent in formal CME outside their workplace during the previous calendar year. Formal CME activities in the Finnish context include symposia, congresses, workshops, or other types of professional meetings with educational agenda. For this report, doctors who had attained their specialist degree before 2005 were included, and they were categorised according to the last specialisation attained. The 49 specialist groups were re-categorised into 15 groups for analytical and confidentiality reasons (Table). The mean reported number of days in CME and 95% confidence intervals were calculated. Differences were further analysed by Poisson regression adjusted for age, gender and working sector (public/private).

## Results

The response rate of the survey was 79.8% (14,168), and 70.2% (7,374) of the surveyed specialists responded to the question about the number of days spent in CME in 2005. The response rate was lowest among the combined group of diagnostic specialties excluding radiologists (60.6%, n = 260) and the highest among specialists in General Practice/Family Medicine (76.4%, n = 1,327) (Table 1). The median age (interquartile range) of the respondents was 49 years (from 44 to 55 years), and 51.7% (3,810) were female. Three quarters of the respondents (75.7%, n = 5,580) were employed in the public sector. The mean reported number of days in CME was 8.8 (95% CI 8.7-9.0). Neurologists and surgery specialists participated in CME the most frequently (10.3 and 10.4 days) and ophthalmologists the least (7.6 days) (Table). In comparison with anaesthesiology and intensive care specialists, most specialists reported having significantly more formal CME, and no group reported having less.

**Table 1:** Participation in formal CME in Finland in 2005 according to specialty group

	Total number of working-age specialists*	Respondents, n (%)	Participation in formal CME, days' mean (95% CI)	IRR (95% CI)**
Anaesthesiology and Intensive Care Medicine	663	430 (64.9)	7.8 (7.4-8.3)	1.00 (reference)
Diagnostic specialties (Clinical Physiology and Nuclear Medicine, Clinical Chemistry, Clinical Microbiology, Clinical Neurophysiology, and Pathology)	429	260 (60.6)	8.6 (7.9-9.3)	1.09 (1.03-1.15)
General Practice/Family Medicine	1,737	1,327 (76.4)	7.8 (7.4-8.1)	0.98 (0.94-1.02)
Neurology	257	191 (74.3)	10.3 (9.3-11.2)	1.32 (1.25-1.39)
Obstetrics and Gynaecology	598	455 (76.1)	9.4 (8.9-10.0)	1.20 (1.15-1.26)
Occupational Health	561	413 (73.6)	7.9 (7.4-8.4)	0.99 (0.94-1.04)
Ophthalmology	396	280 (70.7)	7.6 (7.1-8.2)	0.97 (0.92-1.03)
Other specialties (Phoniatrics, Physical and Rehabilitation Medicine, Geriatrics, Dermatology and Allergology, Clinical Pharmacology and Pharmacotherapy, Sports Medicine, Oncology, Public Health, Forensic Medicine, Clinical Genetics)	750	556 (74.1)	9.4 (8.8-10.0)	1.20 (1.15-1.25)
Otolaryngology	300	215 (71.7)	8.0 (7.4-8.7)	1.02 (0.96-1.08)
Paediatric specialties (Paediatrics and Child Neurology)	592	426 (72.0)	9.6 (9.0-10.3)	1.23 (1.17-1.29)
Psychiatric specialties (Adolescent P., Child P., Forensic P., Psychiatry)	1,234	831 (67.3)	8.9 (8.3-9.5)	1.13 (1.09-1.18)
Radiology	549	340 (61.9)	7.7 (7.2-8.2)	0.98 (0.93-1.03)
Respiratory Medicine and Allergology	194	147 (75.8)	9.4 (8.0-10.7)	1.20 (1.12-1.27)
Specialties related to Internal Medicine (Cardiology, Clinical Haematology, Endocrinology, Gastroenterology, Infectious Diseases, Internal Medicine, Nephrology, Rheumatology)	1,103	783 (71.0)	9.8 (9.5-10.2)	1.26 (1.21-1.31)
Surgical specialties (Cardiothoracic S., Gastroenterologic S., General S., Hand S., Neurosurgery, Oral and Maxillofacial S., Orthopaedics and Traumatology, Paediatric S., Plastic S., Urology, and Vascular Surgery)	1,135	720 (63.4)	10.4 (9.9-10.9)	1.32 (1.27-1.38)
All	10,498	7,374 (70.2)	8.8 (8.7-9.0)	--

CME = Continuing Medical Education, CI = confidence interval, IRR = Incidence Rate Ratio

*Under the age of 63.

**Poisson regression analysis was adjusted for age, gender and working sector (public/private).

Specialties with a small number of physicians were combined in order to maintain confidentiality.

## Discussion

The mean number of days dedicated to formal CME in 2005 was 8.8 per specialist in Finland, a country without a recertification system. Significant variation between specialties in participation in CME was found.

The variation may be explained by differently perceived development needs, varying workload and possibilities to participate, or the availability of suitable CME. For example, shortage of doctors may associate with the increased work load for those at duty and decrease possibilities to participate in external CME activities. We did not have data on participation in other types of professional development activities, such as reading, teaching, taking part in quality work or in regular intra-hospital clinical meetings which might affect the need for external education.

Furthermore, we did not find any reports from other settings on inter-speciality comparisons in participation rates. We therefore suggest further studies on the amount CME in specialist groups, as well as studies to account for variation. Qualitative, rather than quantitative, approaches could give deeper understanding of variation and possible barriers of participation. Studies on the effect of regulations on participation in formal CME are well warranted, though we recognise that participation in formal education accounts for only a part of the continuing professional development of a doctor.

One weakness of the study is that the results are based on subjective estimate on participation, prone to recall bias. However, this will affect all respondents similarly. Due to the high response rate in the specific question (70.2%) the generalisability of the results is good in Finland.

## Conclusions

In Finland in 2005, neurologists and surgery specialists participated in CME the most frequently and ophthalmologists the least. Explanatory studies on this variation and on the effect of the regulatory system on the amount of CME are warranted.

## Competing interests

The authors declare that they have no competing interests.

## Authors' contributions

AHS, JV and MK designed the study. MK and JV carried out the analysis. AHS, JV, HH, TA, JPT and AP interpreted the results. AHS wrote the draft of the manuscript and JV, HH, TA, JPT and AP revised it. All the authors approved the final manuscript.
